# Short- and Long-Term Effects of Subchronic Stress Exposure in Male and Female Brain-Derived Neurotrophic Factor Knock-In Val66Met Mice

**DOI:** 10.3390/biology13050303

**Published:** 2024-04-27

**Authors:** Fernando Antonio Costa Xavier, Silvia Stella Barbieri, Maurizio Popoli, Alessandro Ieraci

**Affiliations:** 1 Laboratory of Molecular and Cellular Biology, Pontifical Catholic University of Rio Grande do Sul, Porto Alegre 90619-900, Brazil; fxavier@pucrs.br; 2 Department of Pharmaceutical Sciences, Università degli Studi di Milano, 20133 Milano, Italy; maurizio.popoli@unimi.it; 3 Unit of Brain-Heart Axis: Cellular and Molecular Mechanisms, Centro Cardiologico Monzino IRCCS, 20138 Milan, Italy; silvia.barbieri@cardiologicomonzino.it; 4 Department of Theoretical and Applied Sciences, eCampus University, 22060 Novedrate, Italy; 5 Department of Neuroscience, Istituto di Ricerche Farmacologiche Mario Negri IRCCS, 20156 Milan, Italy

**Keywords:** stress, fear, neurotrophin, rodents, epigenetic

## Abstract

**Simple Summary:**

Stress is an important risk factor for the pathophysiology of various neuropsychiatric disorders, including anxiety and depression, although people cope differently with adverse life events. While resilient people are able to successfully adapt to stressful experiences, vulnerable people have difficulty coping appropriately with changes in the environment. The reasons for this are not known, but could be explained by individual genetic variants, sex and previous life experiences. The Val66Met variant of Brain-Derived Neurotrophic Factor (BDNF), which impairs intracellular BDNF transport and activity-dependent secretion of BDNF, has been associated with increased susceptibility to the development of various neuropsychiatric disorders, although the evidence is still controversial. Here, we found that exposure to subchronic stress induced an anxiety-like phenotype in all mice, regardless of genotype and sex. Interestingly, all mice are able to recover from the anxiety-like phenotype in the long term, except the male heterozygous mice carrying the Val66Met variant of BDNF. Furthermore, we found that recovery was associated with changes in epigenetic mechanisms and neurotrophic factors. Overall, our results show that the presence of Val66Met BDNF reduces the ability of only male mice to recover from a stressful event.

**Abstract:**

Stress is an important risk factor for the onset of anxiety and depression. The ability to cope with stressful events varies among different subjects, probably depending on different genetic variants, sex and previous life experiences. The Val66Met variant of Brain-Derived Neurotrophic Factor (BDNF), which impairs the activity-dependent secretion of BDNF, has been associated with increased susceptibility to the development of various neuropsychiatric disorders. Adult male and female wild-type Val/Val (BDNF^V/V^) and heterozygous Val/Met (BDNF^V/M^) mice were exposed to two sessions of forced swimming stress (FSS) per day for two consecutive days. The mice were behaviorally tested 1 day (short-term effect) or 11 days (long-term effect) after the last stress session. Protein and mRNA levels were measured in the hippocampus 16 days after the end of stress exposure. Stressed mice showed a higher anxiety-like phenotype compared to non-stressed mice, regardless of the sex and genotype, when analyzed following the short period of stress. In the prolonged period, anxiety-like behavior persisted only in male BDNF^V/M^ mice (*p* < 0.0001). Interestingly, recovery in male BDNF^V/V^ mice was accompanied by an increase in pCREB (*p* < 0.001) and *Bdnf4* (*p* < 0.01) transcript and a decrease in HDAC1 (*p* < 0.05) and *Dnmt3a* (*p* = 0.01) in the hippocampus. Overall, our results show that male and female BDNF Val66Met knock-in mice can recover from subchronic stress in different ways.

## 1. Introduction

Anxiety disorders represent a pervasive and often debilitating class of mental health conditions that affect millions of individuals worldwide. These disorders, characterized by excessive worry, fear and apprehension, can significantly impair daily functioning and quality of life. While the precise etiology of anxiety disorders remains elusive, a growing body of research suggests that a complex interplay of factors, including genetics, neurobiology and environmental stressors, contributes to their development and manifestation [1,2,3]. 

Numerous negative life events, commonly referred to as stress, have been demonstrated to be significant risk factors for anxiety disorders. It is important to keep in mind that stress is a physiological response to environmental change and that the brain has a remarkable ability to adapt and change structurally and functionally in response to stressful experiences. However, the brain’s ability to adapt efficiently to stress varies from person to person and may depend on various factors related to individual differences, such as genetic background, sex, previous life experiences or the type, intensity and duration of stress [3,4,5].

Sex differences have been recognized as a notable factor in the prevalence, presentation and underlying mechanisms of anxiety disorders. Epidemiological studies consistently highlight that females exhibit a higher susceptibility to anxiety disorders compared to males [6,7,8]. However, the exact mechanisms driving these sex differences remain enigmatic and warrant thorough investigation. It is hypothesized that an interplay of hormonal, genetic and environmental factors moderate these sex-specific patterns of susceptibility to anxiety.

Stress is seen as a central trigger for anxiety disorders. The intricate relationship between stress and anxiety involves complex neurobiological cascades that influence emotion processing, cognitive functions and physiological responses [4]. A look at the molecular level reveals a complex interplay of neurotransmitters, hormones and signaling pathways that modulate the stress response. Among these molecular players, brain-derived neurotrophic factor (BDNF) emerges as an important protagonist, which not only modulates neuronal growth and synaptic plasticity, cognitive function and emotional regulation, but also plays a role in stress adaptation and resilience [9,10]. In fact, both acute and chronic stress have been shown to decrease BDNF levels, particularly in the hippocampus and prefrontal cortex, which has been linked to the development of psychiatric and cognitive disorders [11,12].

Remarkably, a common polymorphism called Val66Met, in which a valine is replaced by a methionine at the codon 66, was found only in the human BDNF pro-domain [13]. This genetic variant reduces the activity-dependent release of BDNF [14] and has been correlated with reduced hippocampal volume, learning and memory deficits and augmented susceptibility to stress-related neuropsychiatric disorders [13,15], although these findings have not been confirmed in other studies [16]. This discrepancy may likely be due to the genetic diversity of individuals, age, gender and the various environmental factors to which an individual may be exposed [16]. 

The BDNF^M/M^ knock-in mice recapitulate some of the features described in humans, including impaired hippocampus-dependent memory, an anxiety-like phenotype and reduced hippocampal volume [14]. Remarkably, the heterozygous BDNF^V/M^ mouse, which has no obvious phenotype, shows increased vulnerability to subchronic stress [17,18,19]. However, these studies were only performed on male animals and shortly after the end of stress exposure. Therefore, it is not known whether a similar vulnerability to stress is also present in females and/or if the stress-induced behavioral changes persist over a longer period of time.

Altered transcriptional mechanisms were described in stress-related disorders, such as anxiety and depression [20,21]. The regulation of gene expression is controlled by the synchronized action of epigenetic mechanisms and transcriptional factors. Various enzymes, referred to as writers, erasers and readers, finely regulate epigenetic mechanisms [22,23]. Writers, such as DNA methyltransferases (DNMTs), histone acetyltransferases and histone methyltransferases, add chemical modifications to DNA and histone. Erasers, including histone deacetylases (HDACs), histone demethylases and enzymes that promote DNA demethylation remove these chemical modifications. Readers recognize these epigenetic modifications and translate them into functional consequences [22,23]. 

Altered HDAC and DNMT expression levels have been found in the postmortem brain of patients with psychiatric disorders [24,25], as well as in animal models of stress-related disorders [25,26,27,28,29,30]. Unfortunately, many of the animal model studies have used only males and research into sex-specific differences in transcriptional and epigenetic changes that correlated with subchronic stress exposure is still in its infancy.

The aim of this study was to investigate whether the presence of the human BDNF Val66Met polymorphism in male and female mice can influence the stress-induced behavioral alterations in the short and long term and to investigate the possible associated molecular mechanisms.

## 2. Materials and Methods

### 2.1. Animals

Male and female wild-type mice (BDNF^Val/Val^) and heterozygous mice carrying the human BDNF Val66Met polymorphism (BDNF^Val/Met^) (3–4 months old) were used [14]. The mice were housed under standard conditions (20–22 °C, 12 h light/darkness, light on at 7 am) and given water and food ad libitum. The animal handling and experimental procedures were carried out in compliance with the European Community Council Directive 2010/63/UE and approved by the Italian legislation on animal experimentation (Decreto Legislativo 26/2014, authorization N 349/2015-PR). Every effort was made to minimize animal distress and to reduce the number of animals used in this study.

### 2.2. Forced Swimming Stress (FSS)

Individual mice were placed in a glass beaker (height, 24 cm; diameter, 12 cm) containing 1500 mL water (25 ± 2 °C) for 5 min twice daily for two consecutive days. After FSS, the mice were carefully dried with a towel and returned to their home cage. During the FSS sessions, the mice were video-recorded and total immobility time was evaluated by the software ANY-MAZE 4.99 (Stoelting, purchased from Ugo Basile, Varese, Italy) [26]. One day (short-term effect) or 11 days (long-term effect) after the last stress exposure, mice were evaluated in a battery of behavioral tests. 

### 2.3. Measurement of the Corticosterone Serum Level

Plasma corticosterone levels were measured 30 min after the last stress session. For plasma preparation, trunk blood was collected into an ice-cooled tube containing ethylenediaminetetraacetic acid (0.5 M pH 8.00), separated by centrifugation and stored at 80 °C. A commercial kit (Corticosterone ELISA Kit; Enzo Life Sciences, Florence, Italy) was used to determine the level of corticosterone in plasma [19].

### 2.4. Splash Test

Thirty to sixty minutes before the test, the mice were acclimatized to the room. The mice were sprayed on their backs with a 10% sucrose solution and then placed individually in an empty cage for five minutes. An observer, unaware of the experimental conditions, manually scored and recorded the total time the mice spent grooming during these five minutes. After the test, the mice were immediately placed individually in a normal mouse cage [31].

### 2.5. Novelty-Suppressed Feeding Test

The Novelty-Suppressed Feeding (NSF) Test was conducted as previously described [26]. Briefly, a standard meal pellet was placed on a white paper platform in the center of a large rectangular arena (60 × 40 × 15 cm) filled with wooden bedding. The mice were starved for 16–24 h before the test. A single mouse was placed in the corner of the arena and the latency to bite into the pellet was recorded. Once the first bite was taken, the mice were moved to their home cage where the amount of food ingested over the course of five minutes was weighed (food intake in the home cage) [26].

### 2.6. Sucrose Preference

After the splash test, the mice were housed individually and given two bottles of water for a familiarization period of 24 h. After the NSF test, one of the two bottles was replaced with a bottle containing 1% sucrose for 24 h. After weighing the two bottles, the positions were exchanged for a further 24 h. The test lasted a total of 48 h. The sucrose was calculated by dividing the total amount of sucrose consumed by the total amount of liquid consumed (sucrose plus water) [31].

### 2.7. RNA Isolation and Reverse Transcription

Total mRNA was extracted from the HPC using the Direct-zol RNA MiniPrep (Zymo Research, purchased from Euroclone, Pero, Italy) and the amount was determined by measuring the absorbance at 260 nm with UV spectrophotometry (NanoVue, GE Healthcare Europe GmbH, Milan, Italy). A total of 1 μg of mRNA was reverse-transcribed using the iScript kit (Bio-Rad, Milan, Italy) which contains a blend of oligo(dT) and random hexamer primers [32].

### 2.8. Real-Time PCR

iTaq Universal SYBR Green Supermix (Bio-Rad Laboratories) was used to perform real-time PCR (qPCR) experiments on a CFX Connect Real-Time System (Bio-Rad Laboratories). qPCR conditions were as follows: 2.5 min at 95 °C, 40 cycles of 15 s at 95 °C and 30 s at 60 °C. The amount of relative mRNA was estimated by the comparative CT method using the equation 2^−∆∆CT^ and expressed as fold changes. The primers used for the quantification of each gene are reported in Table 1. Melting curve analysis was performed to verify primers’ specificity. *Gapdh*, *Rps18* and *bAct* were used as reference genes [32].

### 2.9. Western Blot Analysis

Hippocampi were lysed in 0.28 M sucrose (pH 7.4) with phosphatase (Thermo-Fisher Scientific, Milan, Italy) and protease inhibitors (Sigma-Aldrich, Milan, Italy) and centrifuged at 1000× *g* for 5 min. The nucleus enriched in nuclei pellet was resuspended in ice-cold RIPA buffer (15 mM NaCl, 5 mM Tris HCl, pH 7.4, 5 mM EDTA, 1% Triton X-100, 1% sodium deoxycholate and 0.1% SDS) containing phosphatase and protease inhibitors. 15–30 μg of proteins were loaded and separated on SDS-PAGE gels and blotted onto a polyvinylidene difluoride membrane (GE Healthcare Life Sciences, Milan, Italy). The membranes were soaked in 5% milk in Tris Buffer Saline-Tween 20 (TBS-T) and incubated with anti-HDAC1 (1:1000 Cell Signalling Technology, Danvers, MA, USA), anti-HDAC4 (1:1000 Cell Signalling), anti-HDAC5 (1:1000 Santa Cruz Biotechnology, Dallas, TX, USA), cAMP-response element binding protein (CREB) 1:1000 (Cell Signalling), phospho-Ser^133^ CREB 1:2000 (Cell Signalling) and β-actin 1:10,000 (Merck Group). Membranes were washed with TBS-T and then incubated with the corresponding fluorescent IRDye secondary antibody (1:5000, LI-COR Biosciences, Lincoln, NE, USA) or the peroxidase-conjugated secondary antibody (1:5000, Sigma-Aldrich). Peroxidase immunoreactivity bands were revealed by the ECL detection system (Bio-Rad Laboratories, Milan, Italy). Bands were acquired and quantified by the Odyssey LI-COR scanner (LI-COR Biosciences). Total protein levels were normalized for β-actin levels in the same membrane, while protein phosphorylation levels were normalized for the relative total protein [32].

### 2.10. Statistical Analysis

For statistical studies, a two-way analysis (ANOVA) was used, followed by LSD post-hoc for multiple comparisons, when necessary. Statistical analysis was performed using GraphPad Prism 8 (GraphPad Software, La Jolla, CA, USA). Graphical data are presented as mean ± standard error of the mean (SEM).

## 3. Results

### 3.1. The Immobility Time Is Higher in Male BDNF^V/M^ Mice during Exposure to Subchronic FSS

**Figure 1:**
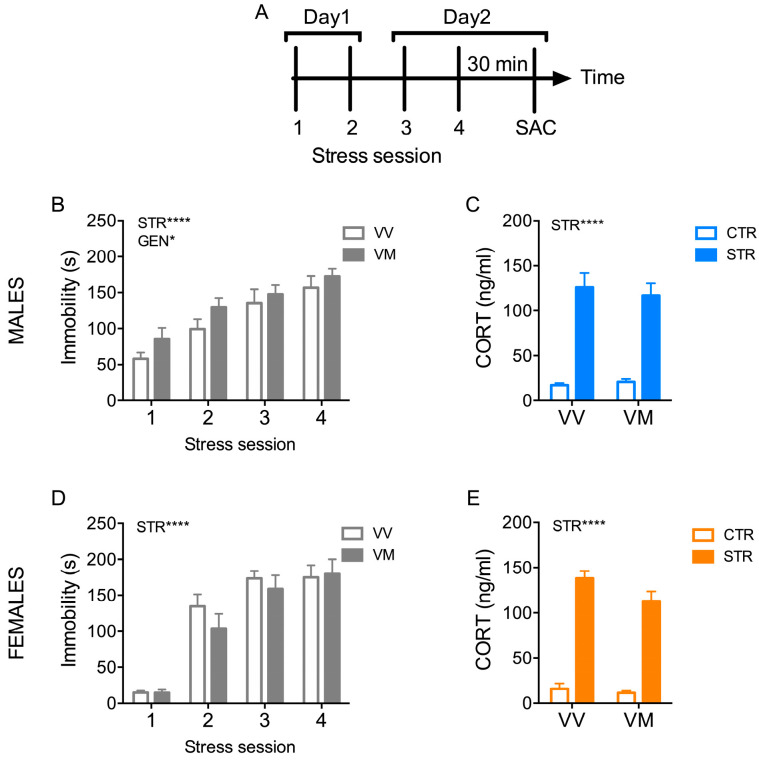
Subchronic FSS increased immobility time more in male BDNF^V/M^ mice. (**A**) Schematic representation of the experimental procedure. (**B**,**D**) Immobility time measured during the 4 sessions of forced swimming stress (FSS) in males (n = 11–12 mice/group) (**B**) and females (n = 13–15 mice/group) (**D**). (**C**,**E**) Plasma corticosterone levels measured 30 min after the last stress exposure in males (**C**) and females (**E**) (n = 11 mice/group). Data are expressed as Mean ± S.E.M. Two-way ANOVA followed by Fisher’s LSD post-hoc analyses. * *p* < 0.05; **** *p* < 0.0001. CTR: Control; STR: stress; VV: BDNF^V/V^; VM: BDNF^V/M^; CORT: corticosterone.

The mice were subjected to a subchronic stress protocol in which they were stressed by forced swimming stress (FSS) for 5 min twice a day for two consecutive days (Figure 1A). In addition to the stress protocol, this paradigm was also used to assess the depressive-like phenotype in both BDNF^V/V^ and BDNF^V/M^ mice, as the period of immobility is considered a direct indicator of a depressive-like phenotype in rodents [33].

The immobility time of mice recorded during the FSS increased during the four stress sessions regardless of genotype and sex (males: stress sessions F_(1, 84)_ = 16.49; *p* < 0.0001; females: stress sessions F_(1, 84)_ = 57.42; *p* < 0.0001). Regarding the genotype factor, there was only a small but significant effect in male mice (Gen F_(1, 84)_ = 4.89; *p* = 0.03), but not in female mice (Gen F_(1, 104)_ =0.95; *p* = 0.33) (Figure 1B,D).

The presence of the BDNF Val66Met polymorphism did not alter the subchronic FSS-induced increase in corticosterone immediately after the last stress session in either male or female mice (males: stress F_(1, 40)_ = 90.46; *p* < 0.0001; females: stress F_(1, 40)_ = 83.02; *p* < 0.0001) (Figure 1C,E).

### 3.2. Subchronic Forced Swimming Stress Induced a Short-Term Anxiety-like Phenotype in Mice

**Figure 2:**
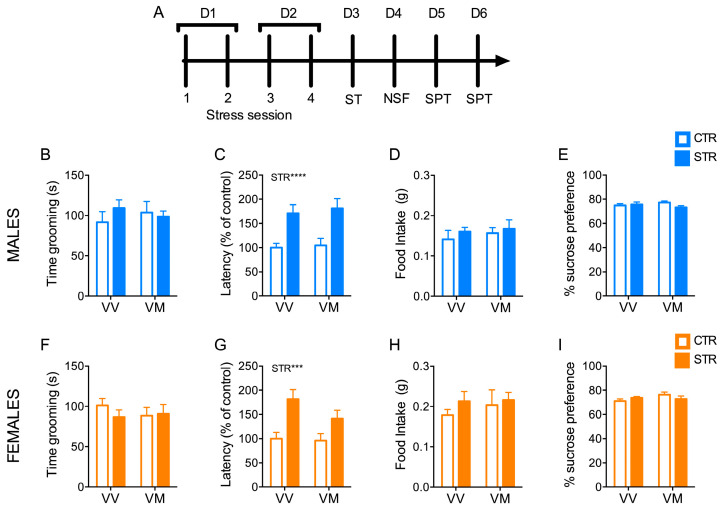
Subchronic FSS induced a short-term anxiety-like phenotype in mice. (**A**) Schematic representation of the experimental procedure. (**B**,**F**) Grooming time measured in the splash test (ST) in male (**B**) and female mice (**F**). (**C**,**G**) Latency to bite the pellet measured during the novelty-suppressed feeding (NSF) in male (**C**) and female mice (**G**). (**D**,**H**) Total amount of food intake in the home cage measured immediately after the NSF test in male (**D**) and female mice (**H**). (**E**,**I**) Sucrose preference test (SPT) in males (**E**) and females (**I**). Data are expressed as Mean ± S.E.M. (males n = 15–17 mice/group; females n = 13–15 mice/group). Two-way ANOVA followed by Fisher’s LSD post-hoc analyses. *** *p* < 0.001; **** *p* < 0.0001. D: day; STR: stress; VV: BDNF^V/V^; VM: BDNF^V/M^.

First, we investigated whether subchronic FSS was able to promote behavioral changes in terms of anxiety- and depressive-like phenotypes in mice from the next day after the last stress exposure (Figure 2A). We found that FSS increased latency time measured in the novelty-suppressed feeding (NSF) in both male and female mice regardless of genotype (stress: males F_(1, 62)_ = 21.00; *p <* 0.0001; females F_(1, 52)_ = 14.92; *p* = 0.0003) (Figure 2C,G). This was not due to a difference in propensity to feed, as there was no difference in total food intake in the home cage measured immediately after the NSF test (Figure 2D,H). In contrast, FSS had no effect on total grooming time as measured by the splash test (Figure 2B,F) and on sucrose preference as measured by the sucrose preference test (SPT) (Figure 2E,I). Overall, these results indicate that FSS induced an anxiety-like, but not a depressive-like phenotype in mice.

### 3.3. Subchronic Forced Swimming Stress Induced a Long-Term Anxiety-like Phenotype Only in Male BDNF^V/M^ Mice

**Figure 3:**
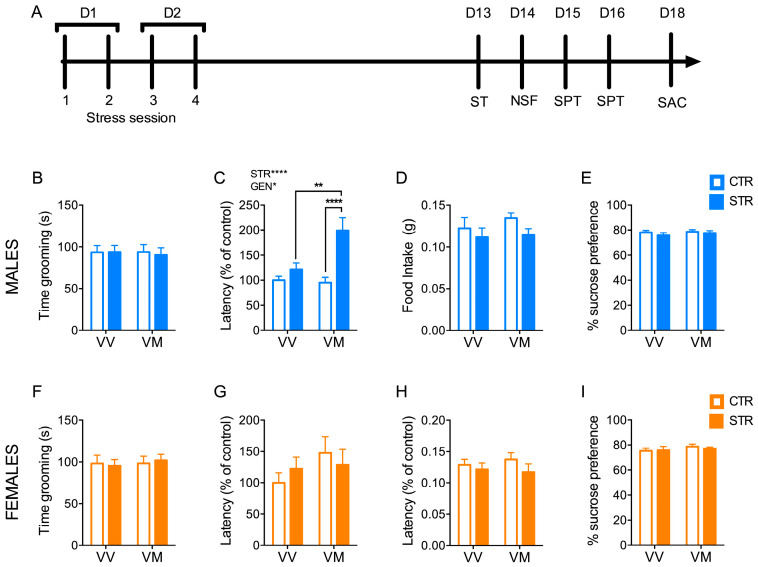
Subchronic FSS induced a long-term anxiety-like phenotype only in male BDNF^V/M^ mice. (**A**) Schematic representation of the experimental procedure. (**B**,**F**) Grooming time measured in the splash test (ST) in male (**B**) and female mice (**F**). (**C**,**G**) Latency to bite the pellet measured during the novelty-suppressed feeding (NSF) in male (**C**) and female mice (**G**). (**D**,**H**) Total amount of food intake in the home cage measured immediately after the NSF test in male (**D**) and female mice (**H**). (**E**,**I**) Sucrose preference test (SPT) in males (**E**) and females (**I**). Data are expressed as Mean ± S.E.M. (males n = 14–18 mice/group; females n = 16–19 mice/group). Two-way ANOVA followed by Fisher’s LSD post-hoc analyses. * *p* < 0.05; ** *p* < 0.01; **** *p* < 0.0001. D: day; STR: stress; GEN: genotype; VV: BDNF^V/V^; VM: BDNF^V/M^.

After observing the development of an anxiety-like phenotype in all stressed mice in the short-term period, we wanted to investigate whether this change persists in the long term or, conversely, if there is a different recovery in the different groups. In the male mice, we found a main effect for stress (F_(1, 63)_ = 14.09; *p* = 0.0004), genotype (F_(1, 63)_ = 4.69; *p* = 0.034) and interaction (F_(1, 63)_ = 6.04; *p* = 0.017) in the latency measured in the NSF test. Notably, stressed BDNF^V/M^ male mice showed a significantly prolonged latency time in the NSF test compared to both control BDNF^V/M^ (*p* < 0.0001) and stressed BDNF^V/V^ (*p* = 0.0013) (Figure 3C). No difference was found in the total amount of food intake in the home cage measured immediately after the NSF test (Figure 3D). In contrast, there were no significant difference among the groups in latency time and in the food intake in the female mice (Figure 3G,H). Like the short-term results, no significant differences in ST and in SPT were found in either males or females (Figure 3B,E,F,I). Overall, these results suggest that the anxiety-like phenotypes promoted by the subchronic FSS persisted only in the male BDNF^V/V^ mice.

### 3.4. Subchronic Forced Swimming Stress Induced a Long-Term Change of BDNF Exons in the Hippocampus in a Sex- and Genotype-Depending Manner

Having found that FSS promoted long-term anxiety-like effects in a sex- and genotype-dependent manner, we next performed qPCR and western blot analyses to determine whether the behavioral changes were associated with molecular/biochemical alterations in the hippocampus.

**Figure 4:**
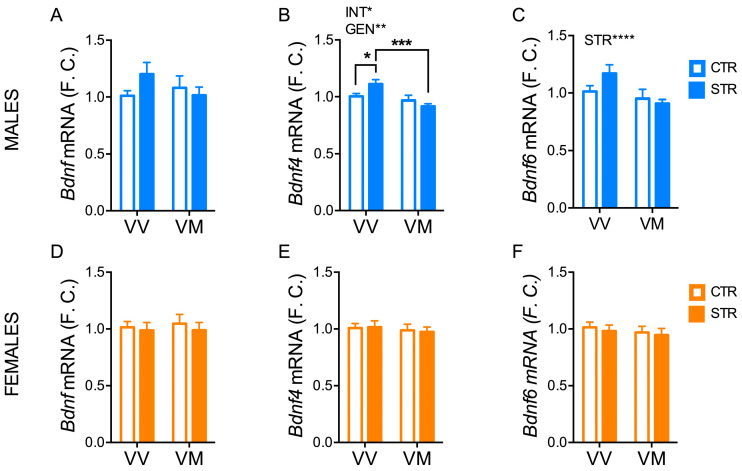
Subchronic FSS induced a long-term change of BDNF exons in the hippocampus in a sex- and genotype-depending manner. (**A**–**F**) mRNA of total *Bdnf* (**A**–**D**) and *Bdnf4* (**B**–**E**) and *Bdnf6* (**C**–**F**) exons measured by qPCR in the hippocampus of male and female mice 16 days after the last stress exposure. Data are expressed as Mean ± S.E.M. (males n = 14–18 mice/group; females n = 16–19 mice/group). Two-way ANOVA followed by Fisher’s LSD post-hoc analyses. * *p* < 0.05; ** *p* < 0.01; *** *p* < 0.001; **** *p* < 0.0001. INT: interaction; GEN: genotype; VV: BDNF^V/V^; VM: BDNF^V/M^.

In males, mRNA levels of exon *Bdnf4* and *Bdnf6* transcripts in the hippocampus were overall lower in BDNF^V/M^ mice compared to BDNF^V/V^ mice (Gen: *Bdnf4* F_(1, 47)_ = 11.69; *p* = 0.0013; *Bdnf6* F_(1, 47)_ = 11.81; *p* = 0.013) (Figure 4B,C). Notably, FSS had a different effect on *Bdnf4* levels in the two genotypes (Int: F_(1, 47)_ = 5.61; *p* = 0.022). Indeed, *Bdnf4* levels in the hippocampus of stressed BDNF^V/V^ mice were higher compared to both BDNF^V/V^ control (*p* < 0.05) and BDNF^V/M^ stressed mice (*p* < 0.001) (Figure 4B). No significant differences were found among the groups for the total *Bdnf* levels in males. Moreover, no differences were found for all *Bdnf* exons analyses in females (Figure 4D–F).

### 3.5. Subchronic Forced Swimming Stress Induced a Long-Term Change of HDACs in the Hippocampus in a Sex- and Genotype-Depending Manner

**Figure 5:**
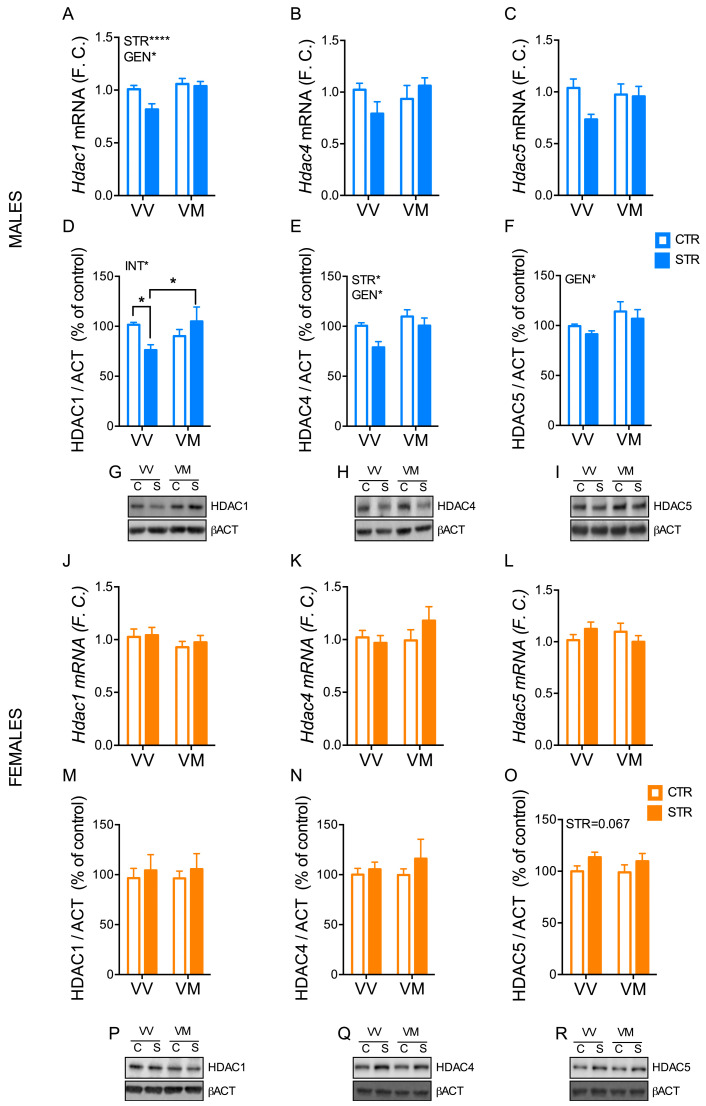
Subchronic FSS induced a long-term change of HDACs in the hippocampus in a sex- and genotype-depending manner. (**A**–**C**,**J**–**L**) mRNA of total *Hdac1* (**A**,**J**) and *Hdac4* (**B**,**K**) and *Hdac5* (**C**,**L**) exons measured by qPCR in the hippocampus of male and female mice 12 days after the last stress exposure. Data are expressed as Mean ± S.E.M. (males n = 12–14 mice/group; females n = 11–12 mice/group). (**D**–**F**,**M**–**O**) Protein levels of nuclear HDAC1 (**D**,**M**) and HDAC4 (**E**,**M**,**N**) and HDAC5 (**F**,**O**) evaluated by western blot in the hippocampus of male and female mice 16 days after the last stress exposure. (**G**–**I**,**P**–**R**) Representative western blot images. Data are expressed as Mean ± S.E.M. (males n = 8–9 mice/group; females n = 6 mice/group). Two-way ANOVA followed by Fisher’s LSD post-hoc analyses. * *p* < 0.05; **** *p* < 0.0001. INT: interaction; GEN: genotype; VV: BDNF^V/V^; VM: BDNF^V/M^; C: control; S: stress. Full western blot figures can be found in Supplementary Material.

While mRNA levels of *Hdac1* in male mice were affected by both stress (F_(1, 47)_ = 7.78; *p* = 0.032) and genotype (F_(1, 47)_ = 12.88; *p* = 0.007) (Figure 5A), protein levels only showed a main interaction effect between genotype and stress (F_(1, 29)_ = 5.74; *p* = 0.023) (Figure 5D,G). Indeed, HDAC1 protein levels were significantly reduced in stressed BDNF^V/V^ mice compared to both BDNF^V/V^ control (*p* < 0.05) and BDNF^V/M^ stressed mice (*p* < 0.05) (Figure 5D,G). In addition, HDAC4 and HDAC5 protein levels were differentially expressed overall in BDNF^V/V^ mice compared to BDNF^V/M^ mice (HDAC4: F_(1, 29)_ =6.32; *p* = 0.017; HDAC5: F_(1, 29)_ = 4.47; *p* = 0.043), while FSS only affected HDAC4 levels in the long-term period (F_(1, 29)_ = 6.23; *p* = 0.019) (Figure 5E,F,H,I). No significant differences were found among the groups for mRNA levels of *Hdac4* and *Hdac5* in males (Figure 5B,C). Furthermore, we only found a trend for stress effect for HDAC5 protein levels (F_(1, 20)_ = 3.74; *p* = 0.067) and no significant differences for all other HDACs analyses in females (Figure 5J–R). 

### 3.6. Subchronic Forced Swimming Stress Induced a Long-Term Change of Dnmt in the Hippocampus in a Sex- and Genotype-Depending Manner

**Figure 6:**
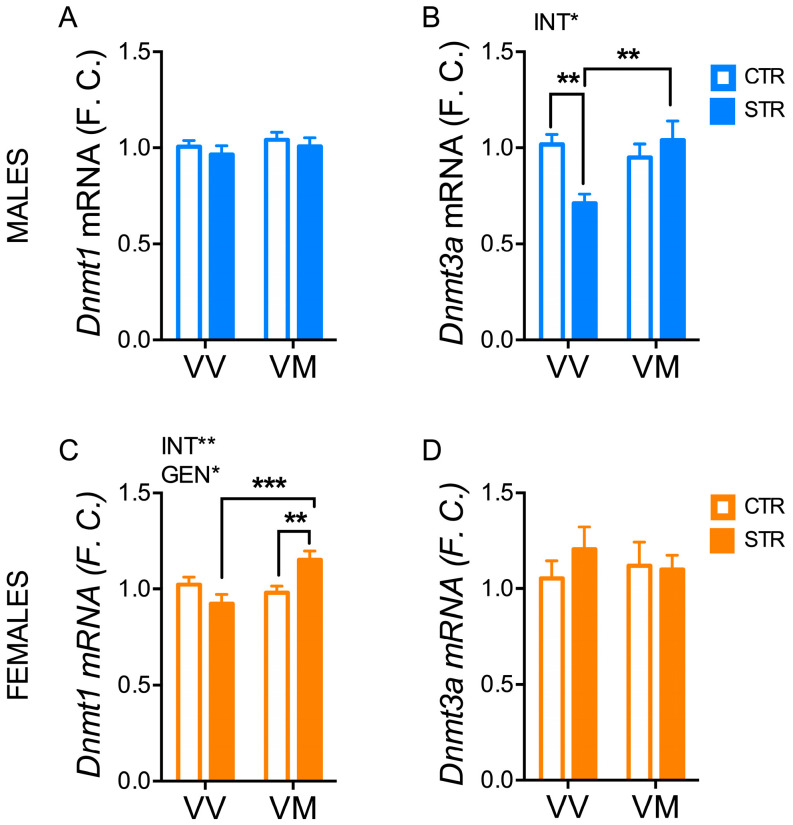
Subchronic FSS induced a long-term change of Dnmts in the hippocampus in a sex- and genotype-depending manner. (**A**–**D**) mRNA of total *Dnmt1* (**A**,**C**) and *Dnmt3a* (**B**,**D**) measured by qPCR in the hippocampus of male and female mice 16 days after the last stress exposure. Data are expressed as Mean ± S.E.M. (males n = 12–14 mice/group; females n = 11–12 mice/group). Two-way ANOVA followed by Fisher’s LSD post-hoc analyses. * *p* < 0.05; ** *p* < 0.01; *** *p* < 0.001. INT: interaction; GEN: genotype; VV: BDNF^V/V^; VM: BDNF^V/M^.

We found a main interaction effect between stress and genotype for *Dnmt3a* in males (F_(1, 47)_ = 7.14; *p* = 0.01) (Figure 6B), whereas no significant differences were observed for *Dnmt1* in male mice (Figure 6A). In particular, FSS reduced hippocampal *Dnmt3a* mRNA levels in male BDNF^V/V^ mice (*p* < 0.01), but not in male BDNF^V/M^ mice (*p* > 0.05) (Figure 6B). In contrast, *Dnmt1* mRNA levels showed a main effect for genotype (F_(1, 42)_ = 4.66; *p* = 0.037) and the interaction between stress and genotype (F_(1, 42)_ = 9.81; *p* = 0.003) (Figure 6C), while no significant differences were found for *Dnmt3a* in female mice (Figure 6D). Notably, FSS promoted the upregulation of *Dnmt1* mRNA levels in the hippocampus of female BDNF^V/M^ mice (*p* < 0.01), but not in female BDNF^V/V^ mice (*p* > 0.05) (Figure 6C).

### 3.7. Subchronic Forced Swimming Stress Induced a Long-Term Change of CREB Phosphorylation in the Hippocampus in a Sex- and Genotype-Depending Manner

**Figure 7:**
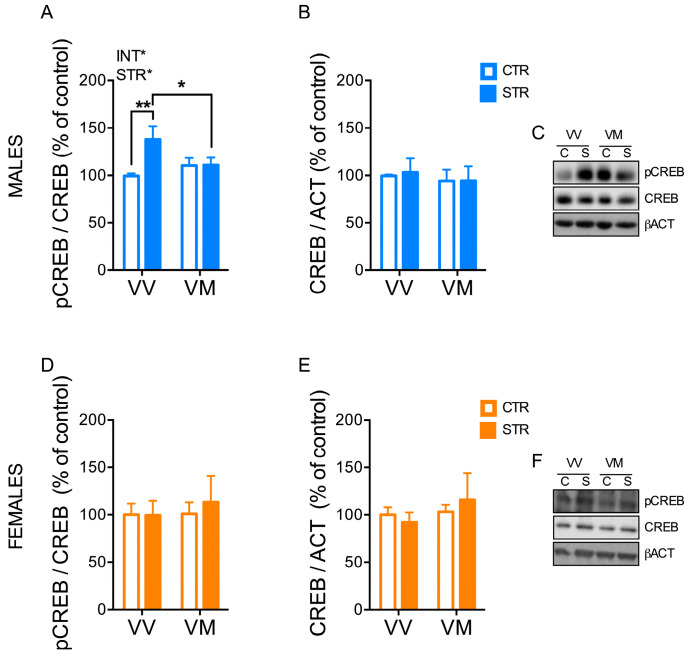
Subchronic FSS induced a long-term change of CREB phosphorylation in the hippocampus in a sex- and genotype-depending manner. (**A**–**F**) Quantification of protein levels of nuclear pCREB (**A**,**D**) and CREB (**B**,**E**) evaluated by western blot in the hippocampus of male and female mice 16 days after the last stress exposure. (**C**,**D**) Representative western blot images. Data are expressed as Mean ± S.E.M. (males n = 8–9 mice/group; females n = 6 mice/group). Two-way ANOVA followed by Fisher’s LSD post-hoc analyses. * *p* < 0.05; ** *p* < 0.01. INT: interaction; STR: stress; VV: BDNF^V/V^; VM: BDNF^V/M^; C: control; S: stress.

The levels of phosphorylated CREB in males were affected by stress (F_(1, 29)_ = 4.76; *p* = 0.037) and the interaction between stress and genotype (F_(1, 29)_ = 4.76; *p* = 0.042) (Figure 7A,C), while no difference was found for the total amount of CREB (Figure 7B,C). Of note, phosphorylated CREB levels were higher in the stressed male BDNF^V/V^ mice than in the control male BDNF^V/V^ mice (*p* < 0.01) and the stressed male BDNF^V/M^ mice (*p* < 0.05) (Figure 7A). No significant difference was found for the phosphorylated CREB and total CREB levels in female mice (Figure 7D–F).

## 4. Discussion

In this study, we found that FSS induces a short-term anxiety-like phenotype in mice, independent of sex or genotype, and that this phenotype persists after 12 days only in male BDNF^V/M^ mice. The recovery of the anxiety-like phenotype in male BDNF^V/V^ mice is associated with a greater long-term decrease in HDAC1 and *Dnmt3a* levels and a greater increase in pCREB and *Bdnf4* levels in the hippocampus. In contrast, in females, there was a significant increase in *Dnmt1* only in the stressed BDNF^V/M^ mice.

A previous study reported that a 7-day restraint stress paradigm induced a short-term depression- and anxiety-like phenotype only in male BDNF^V/M^ mice, but not in male BDNF^V/V^ mice [17], indicating that the presence of the Met allele may increase susceptibility to subchronic stress. In addition, another study reported that subchronic variable stress promotes a short-term anxiety- and depressive-like phenotype in female mice but not in male mice, suggesting that female mice are more susceptible to subchronic stress [31], although this was not confirmed in another study [34]. Here, we reported that FSS can induce a short-term anxiety-like phenotype in all stressed mice, regardless of genotype or sex. Our results appear to contrast with some of the previously published data, which may be due to the different stress paradigm we used, suggesting that subchronic FSS is more intense than other types of subchronic stress. Remarkably, we have shown here, for the first time, that the anxiety-like phenotype is only maintained in male BDNF^V/M^ mice after 12 days, suggesting that all mice except male BDNF^V/M^ mice have the ability to recover the phenotype. As far as we know, the long-term consequences of subchronic stress exposure have not yet been investigated. On the other hand, several studies have looked at the long-term consequences of acute stress and have come to different conclusions depending on the type of stress, the gender and the time spent after the last stress exposure was investigated [35,36,37,38,39,40]. In future studies it would be interesting to investigate whether the male BDNF^V/M^ mice require a longer time to recover from the stress-induced anxiety-like phenotype or whether it is a permanent behavioral disorder.

Several experimental pieces of evidence have shown that stress can induce epigenetic changes and that these mechanisms are related to the development of some neuropsychiatric disorders such as anxiety and depression. In particular, it has been found that an increase in the levels of certain HDAC isoforms is associated with the development of an anxiety-like phenotype, while a reduction in these isoforms can reverse or prevent this phenotype [26,29,41,42]. Our data are consistent with these findings, as we found a greater decrease in HDAC1 in the hippocampus of BDNF^V/V^ mice than in BDNF^V/M^ mice, which was associated with the disappearance of the anxiety-like phenotype only in BDNF^V/V^ mice. Thus, a decrease in this enzyme could promote histone acetylation and therefore the transcription of certain genes, including BDNF, thus inducing an adaptive response to stress. This result could confirm the greater recovery capacity of the male V/V genotype, while these mechanisms do not seem to be involved in the persistence of the anxiety-like phenotype in the male V/M mice in the long-term analysis.

In contrast, the nuclear expression of HDAC4 is significantly reduced by stress in both genotypes. Some evidence in the literature suggests that a reduction in HDAC4 levels may have a specific effect on the anxiety-like phenotype; in particular, downregulation of HDAC4 expression has been shown to prevent the development of the anxiety-like phenotype in stressed mice [29]. A long-term decrease in these levels could indicate a protective response that is activated to counteract the effects of stress. Our results showing a similar decrease in HDAC4 in the two genotypes may suggest that the decrease in this enzyme alone is not sufficient to reverse the anxious-like phenotype, but that other mechanisms are required, such as a decrease in HDAC1 and an increase in pCREB, which were only detected in BDNF^V/V^ mice.

Furthermore, we observed a long-term increase in pCREB only in male BDNF^V/V^-stressed mice. Several studies have linked pCREB expression to anxiolytic and antidepressant effects. For example, treatment with an antidepressant such as fluoxetine, or with anxiolytics such as rolipram or zolpidem, can increase pCREB levels, suggesting that an increase in pCREB may promote anxiolytic activity [43,44,45]. In addition, chronic administration of BDNF in mice was found to cause an increase in pCREB in the hippocampus, which has an antidepressant effect [46]. Taken together, this information may therefore suggest that the BDNF^V/V^ genotype is capable of activating long-term stress response processes that lead to an increase in pCREB, which promotes recovery of the anxiety-like phenotype and exerts an anxiolytic and antidepressant effect.

Our results showing that the recovery from the anxiety-like phenotype in male BDNF^V/V^ is associated with a reduction in *Dnmt3a* mRNA levels in the hippocampus are consistent with previous findings showing increased DNMT3a expression and activity in various brain regions [31,47,48]. Moreover, pharmacological or molecular lowering of DNMT3a levels correlated with a rescue of stress-induced behavioral impairments [31,49]. The specific upregulation of *Dnmt1* in the hippocampus of stressed female BDNF^V/M^ mice is quite unexpected and requires further investigation.

At present, the causes of the different response in female and male BDNF^V/M^ heterozygous mice have not yet been identified. One of the factors that could explain this different response in female and male heterozygous mice could be due to the effect of female sex hormones, which can mutually interact with BDNF and its receptor TrkB, directly inducing BDNF expression [50]. In fact, it has been observed that TrkB is in the phosphorylated (i.e., active) state mainly when estradiol concentrations are high (proestrus); this may also result in increased intracellular Ca^2+^ levels and subsequent overexpression of CREB and pCREB. In addition, Bath et al. [51] showed that, in female mice carrying the Met allele, anxious behavior showed significant variations depending on the estrus phase; in particular, a significant increase in anxious behavior was reported during the estrus phase. In our studies, we did not examine the estrus phase of females during the behavioral tests and at the time of killing. It is possible that opposing effects during different phases of the cycle may have masked any subchronic stress effects. In future studies, it would be interesting to investigate (1) whether a two-day FSS exposure is able to induce long-term behavioral/molecular changes dependent on the estrus cycle and (2) whether a period longer than two days of FSS exposure could promote behavioral/molecular changes in female mice and whether this might depend on the Val66Met polymorphism of BDNF.

## 5. Conclusions

In summary, this work has shown that a subchronic stress protocol can elicit different long- and short-term responses between wild-type mice and mice carrying the human Val66Met polymorphism of BDNF in a sex-dependent manner. While in male BDNF^V/M^ mice the anxiety-like phenotype persisted for several days after FSS exposure, phenotype recovery in male BDNF^V/V^ mice was accompanied by a decrease in HDAC1 and *Dnmt3a* and an increase in pCREB and *Bdnf4*. Although all female mice also recovered from the anxiety-like phenotype in the long term, the molecular changes observed in males were not detected in female mice, suggesting that females utilize different mechanisms to compensate. Future studies will be required to better investigate the mechanisms involved in the anxiety-like phenotype and the different mechanisms of recovery from stress exposure in wild-type and heterozygous males and females to identify potential new targets for the treatment of neuropsychiatric disorders.

## Figures and Tables

**Table 1:** List of primers.

Gene	Primer Forward	Primer Reverse
*Bdnf*	TCGTTCCTTTCGAGTTAGCC	TTGGTAAACGGCACAAAAC
*Bdnf4*	CAGAGCAGCTGCCTTGATGTTT	CGCCTTCATGCAACCGAAGTAT
*Bdnf6*	ACAATGTGACTCCACTGCCGG	CGCCTTCATGCAACCGAAGTAT
*Hdac1*	GAGTTCTGTCAGTTGTCCACGG	TTCAGACTTCTTTGCATGGTGC
*Hdac4*	CAATCCCACAGTCTCCGTGT	CAGCACCCCACTAAGGTTCA
*Hdac5*	TGTCACCGCCAGATGTTTTG	TGAGCAGAGCCGAGACACAG
*Dnmt1*	GGACACAGGTGCCCGCGA	ATGAACCCCAGATGTTGACCA
*Dnmt3a*	AGATCATGTACGTCGGGGAC	CAATCACCAGGTCGAATGGG
*Rps18*	TGGAGCGAGTGATCACCATCA	CCTCACGCAGCTTGTTGTCTA
*bAct*	GCCAGAGCAGTAATCTCCTTCT	AGTGTGACGTTGACATCCGTA
*Gapdh*	CGTGCCGCCTGGAGAAACC	CGTGCCGCCTGGAGAAACC

## Data Availability

The data that support the findings of this study are available from the corresponding author upon reasonable request.

## Supplementary Materials

The following supporting information can be downloaded at: https://www.mdpi.com/article/10.3390/biology13050303/s1, Western blot figures.
