# Supplementary figures and images for: Short- and Long-Term Effects of Subchronic Stress Exposure in Male and Female Brain-Derived Neurotrophic Factor Knock-In Val66Met Mice
Source: Biology (Basel). 2024 Apr 27;13(5):303. doi: 10.3390/biology13050303 (PMC11118886; doi:10.3390/biology13050303)

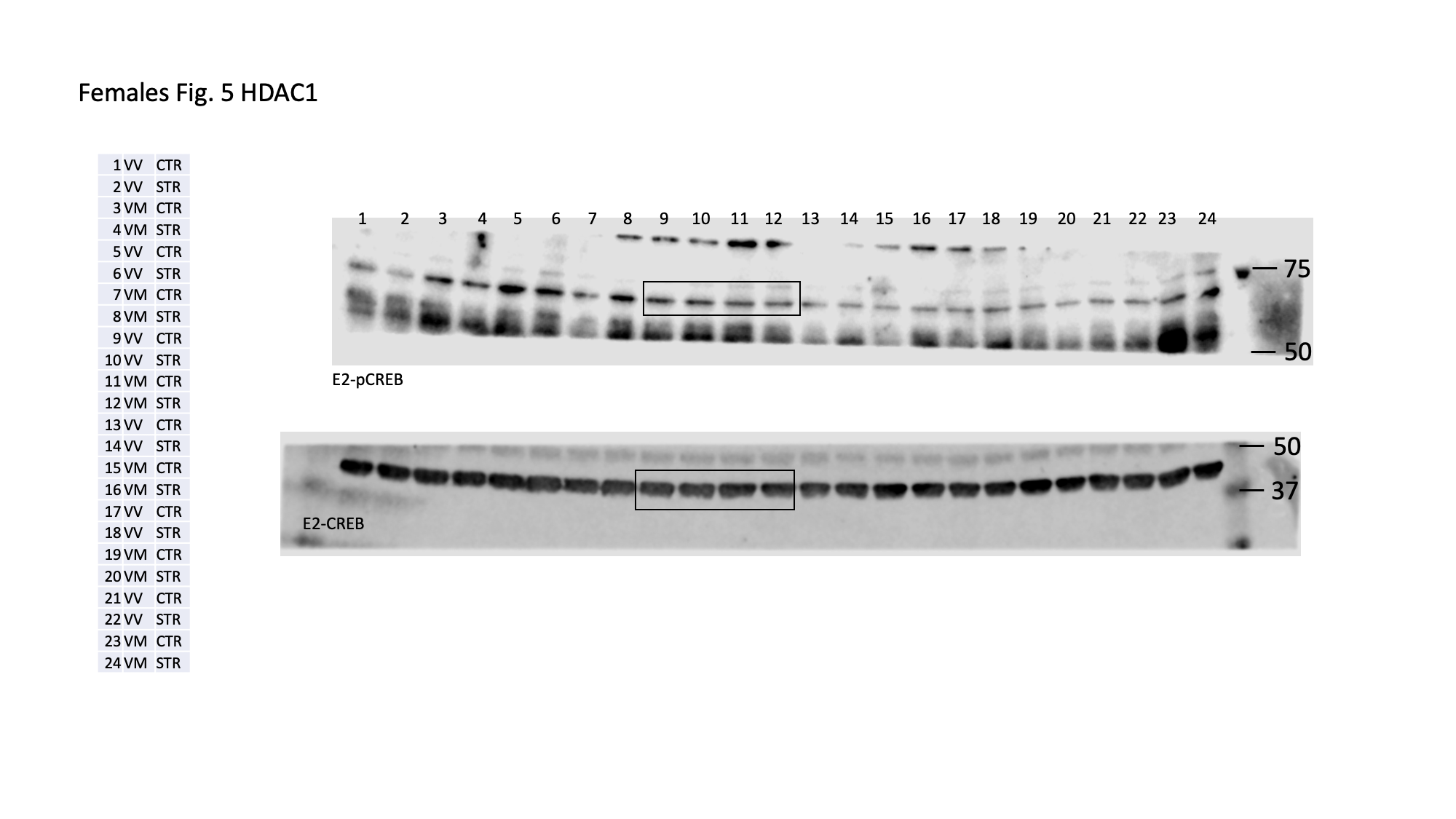

Supplement: Supplementary file 1 [file biology-13-00303-s001.zip › Fig5_HDAC1_Females.tiff]

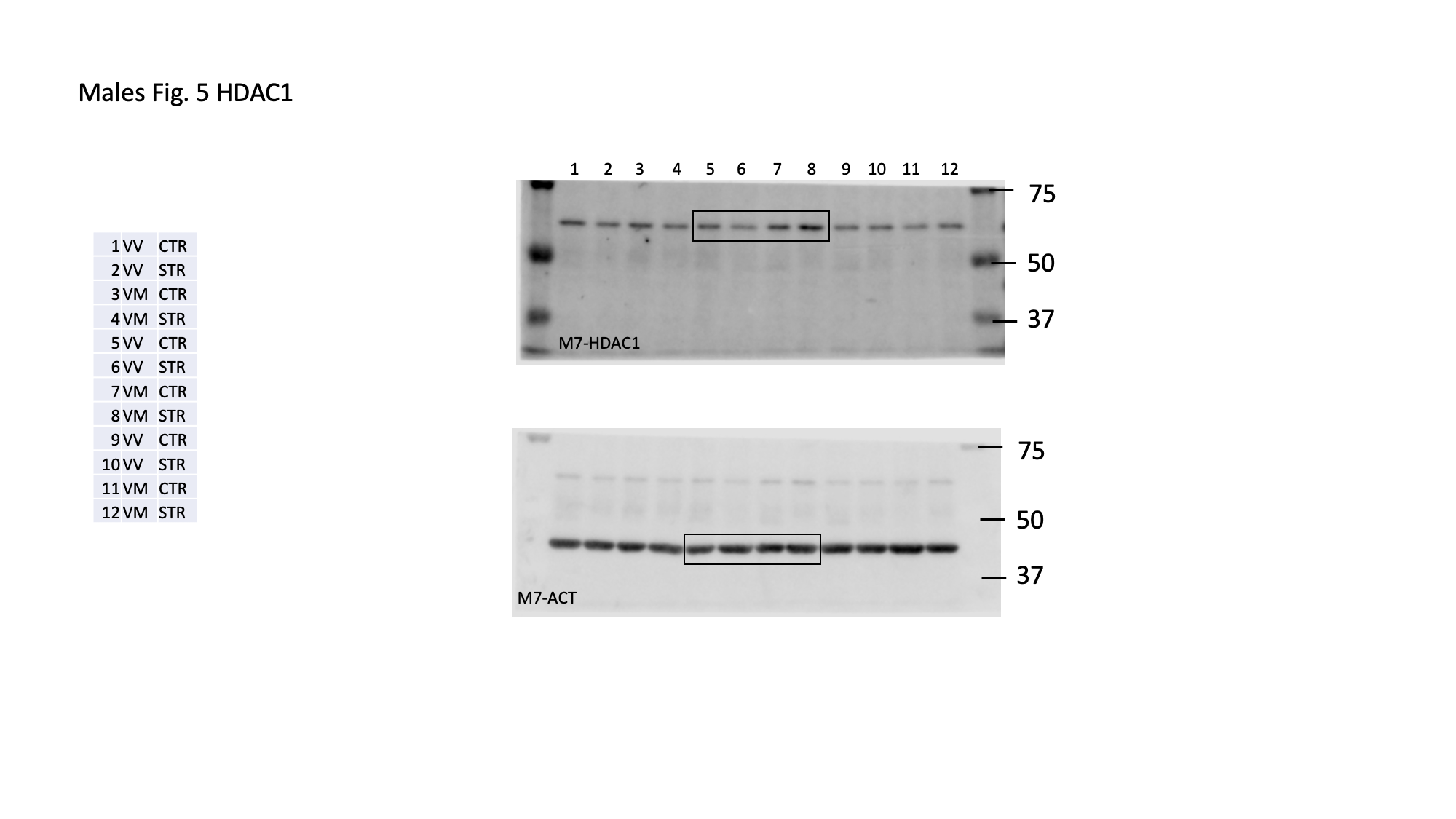

Supplement: Supplementary file 1 [file biology-13-00303-s001.zip › Fig5_HDAC1_Males.tiff]

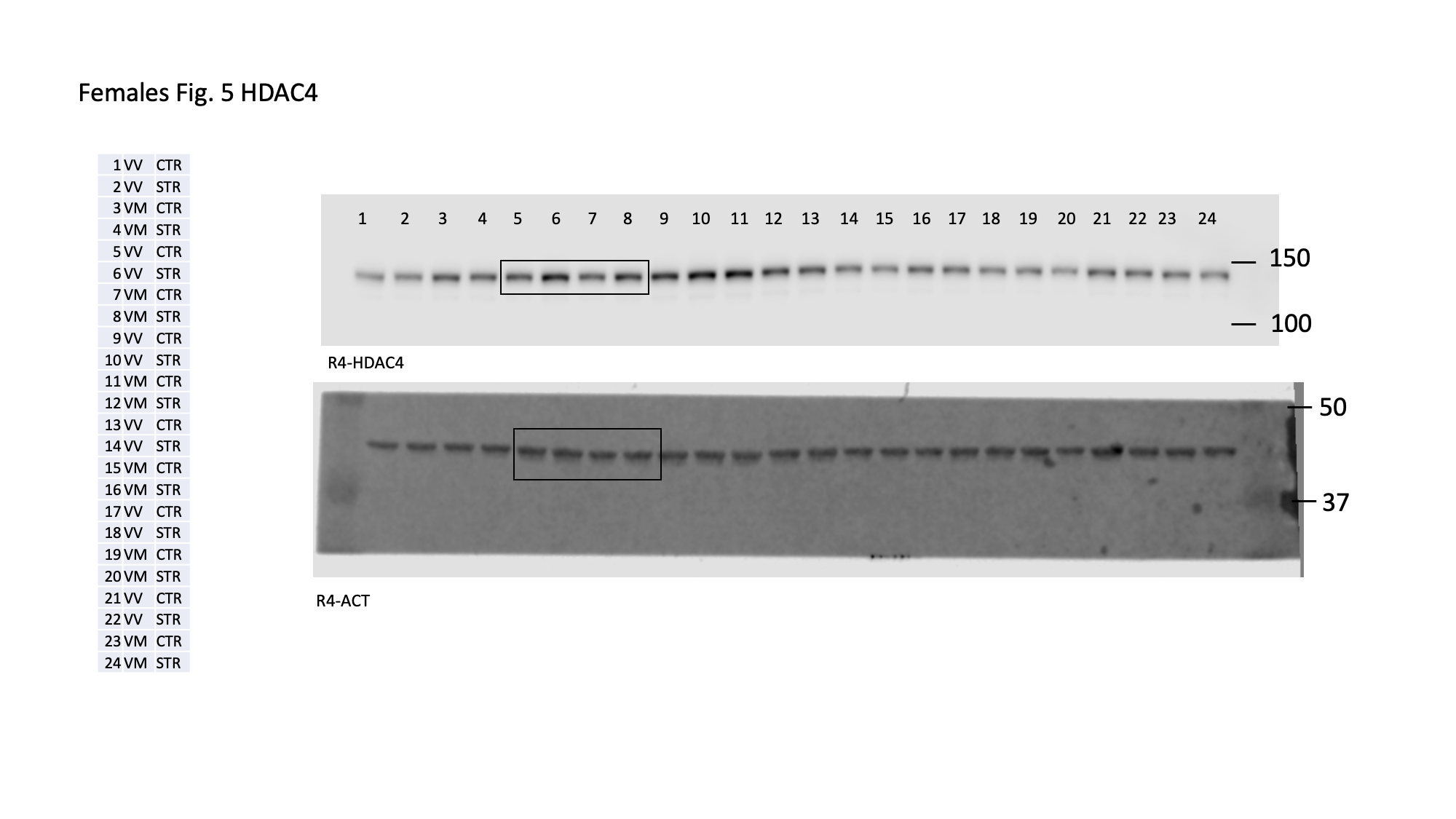

Supplement: Supplementary file 1 [file biology-13-00303-s001.zip › Fig5_HDAC4_Females.tiff]

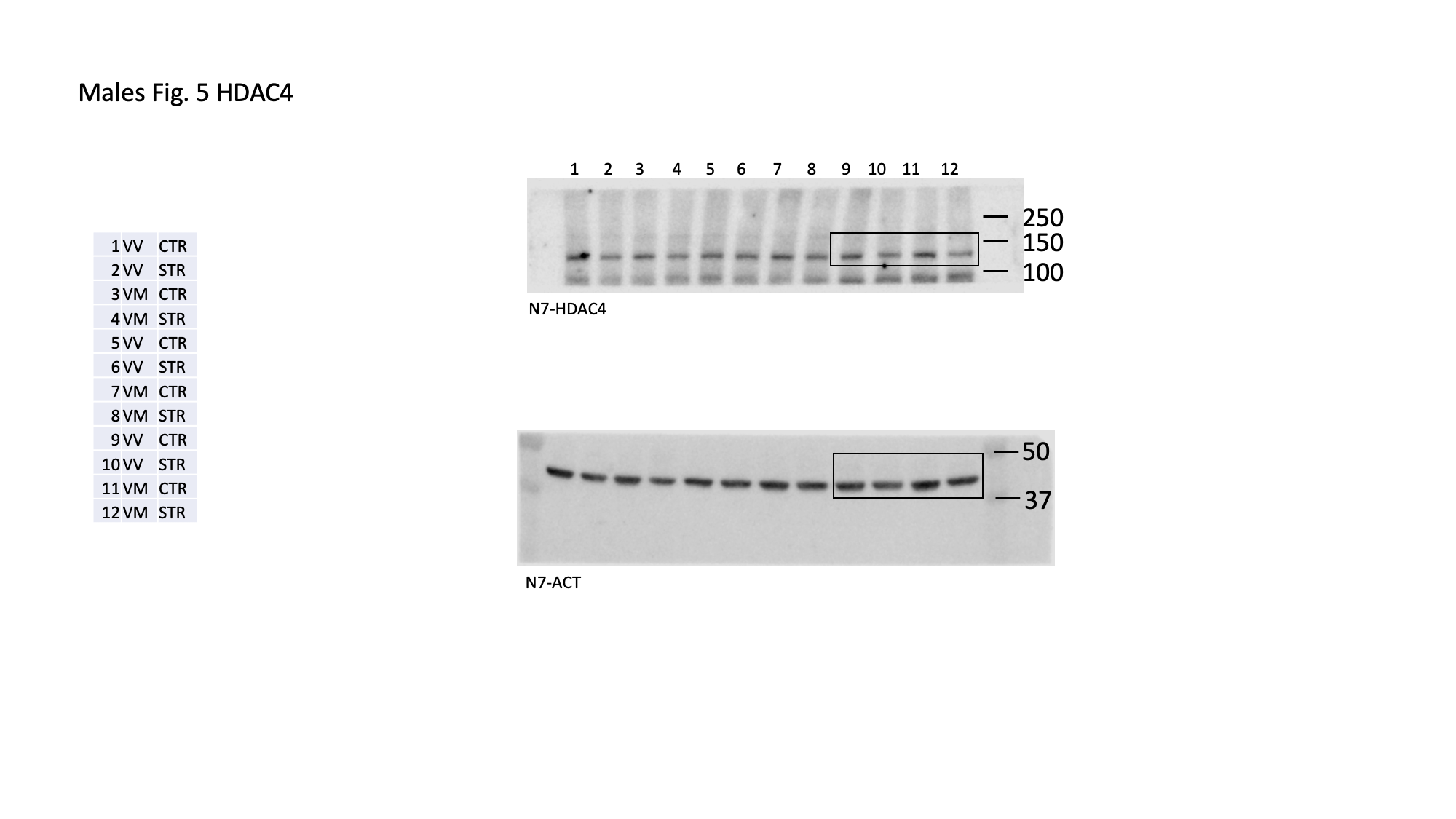

Supplement: Supplementary file 1 [file biology-13-00303-s001.zip › Fig5_HDAC4_Males.tiff]

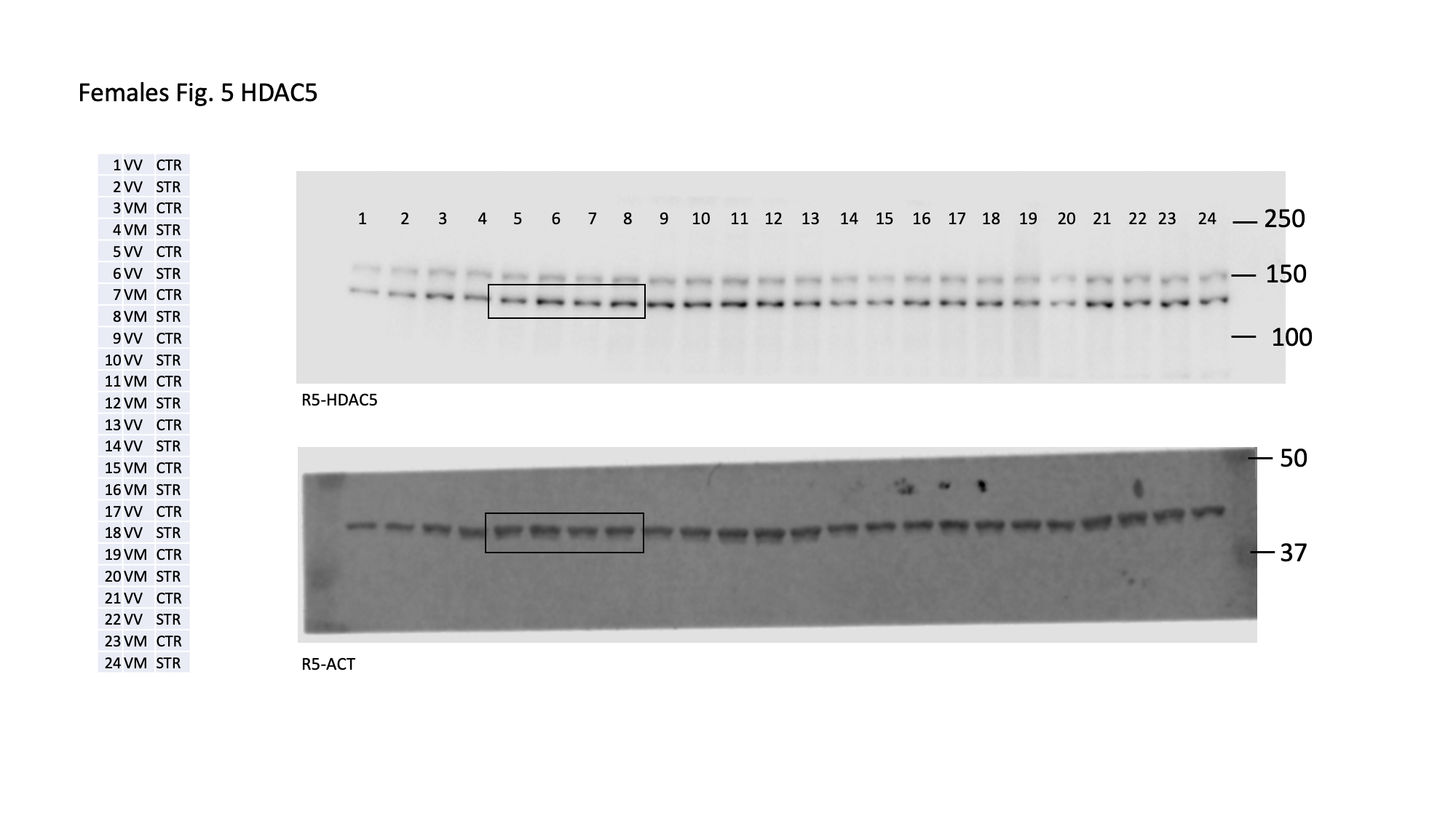

Supplement: Supplementary file 1 [file biology-13-00303-s001.zip › Fig5_HDAC5_Females.tiff]

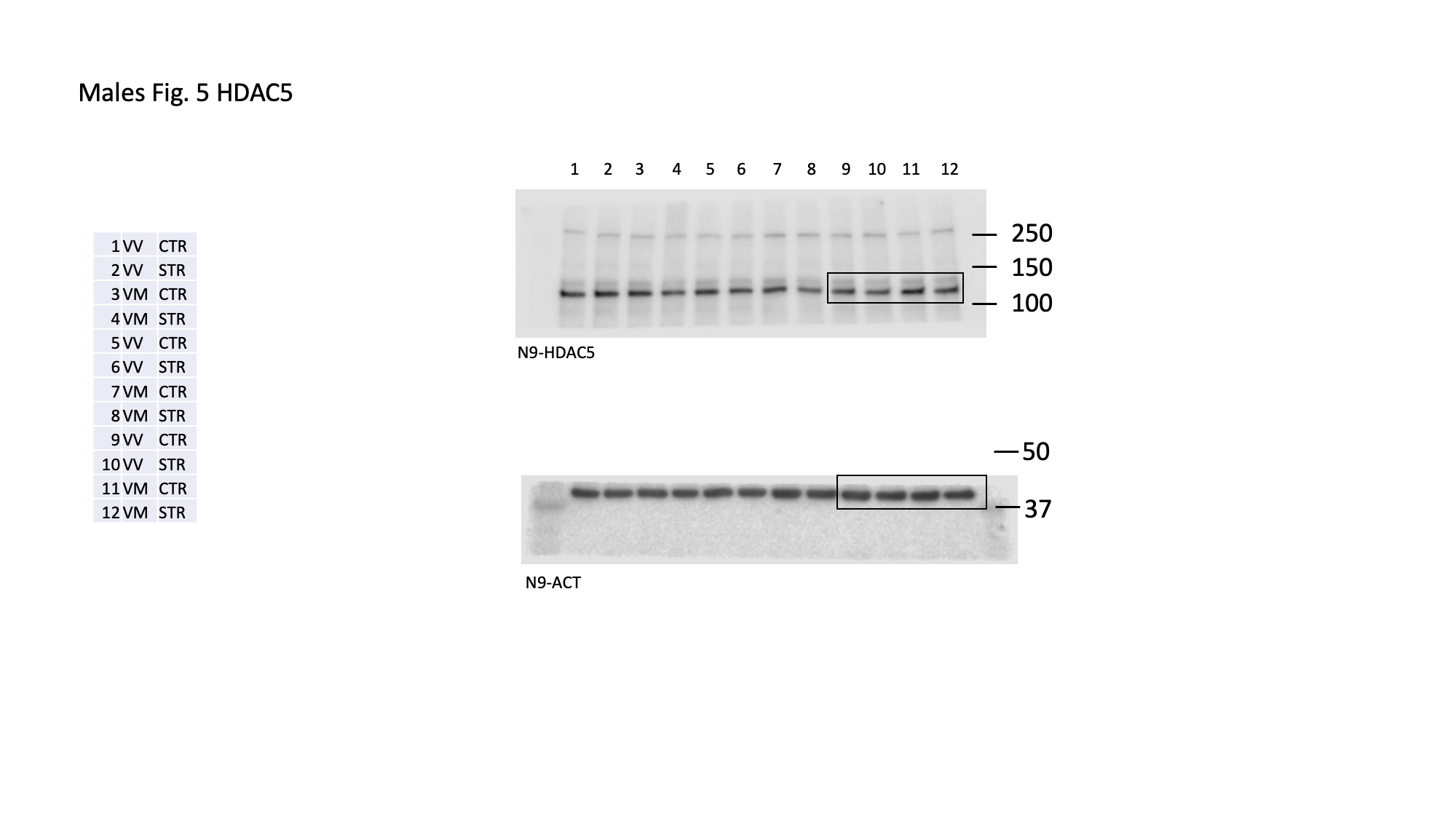

Supplement: Supplementary file 1 [file biology-13-00303-s001.zip › Fig5_HDAC5_Males.tiff]

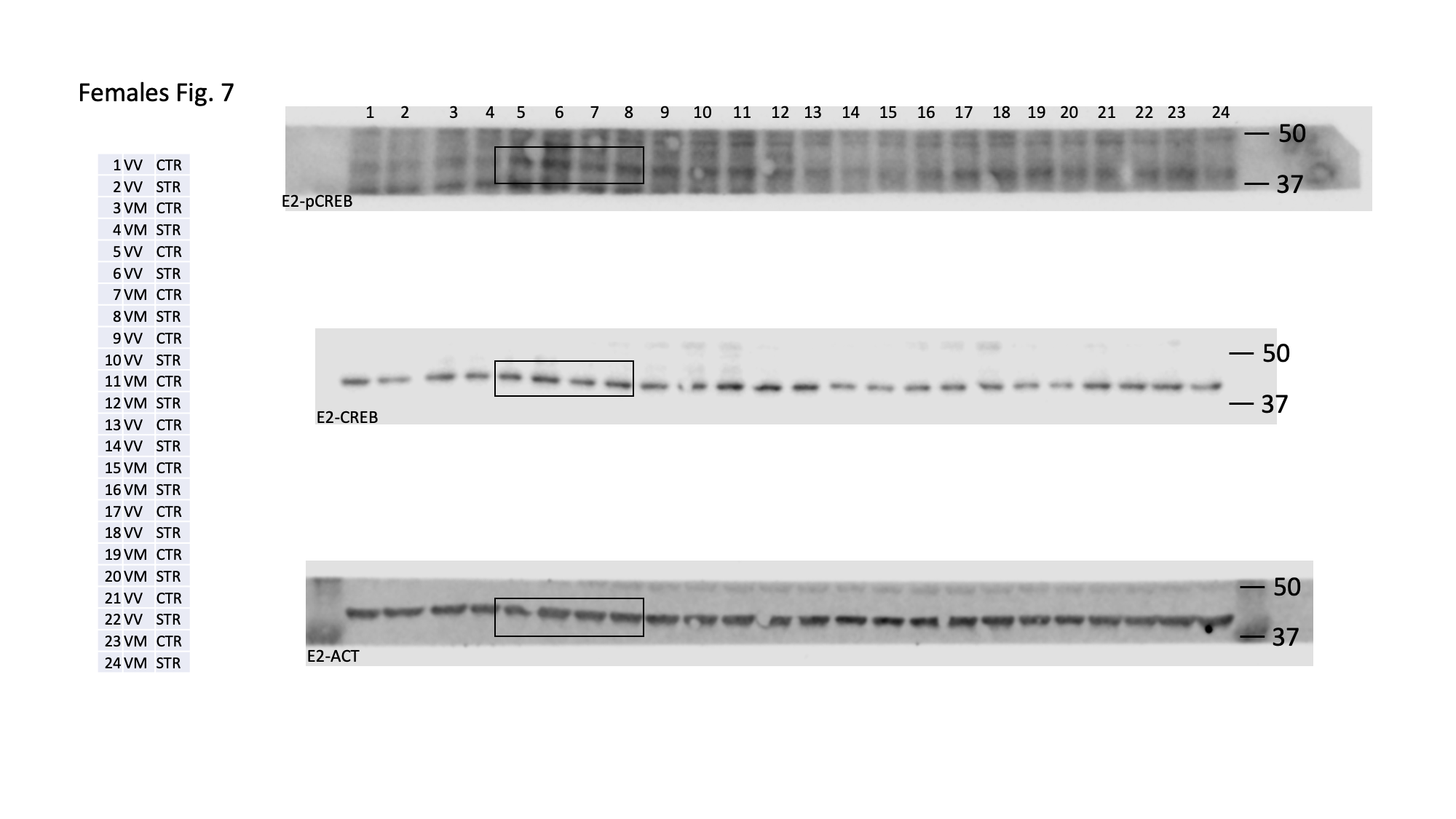

Supplement: Supplementary file 1 [file biology-13-00303-s001.zip › Fig7_pCREB-CREB_Females.tiff]

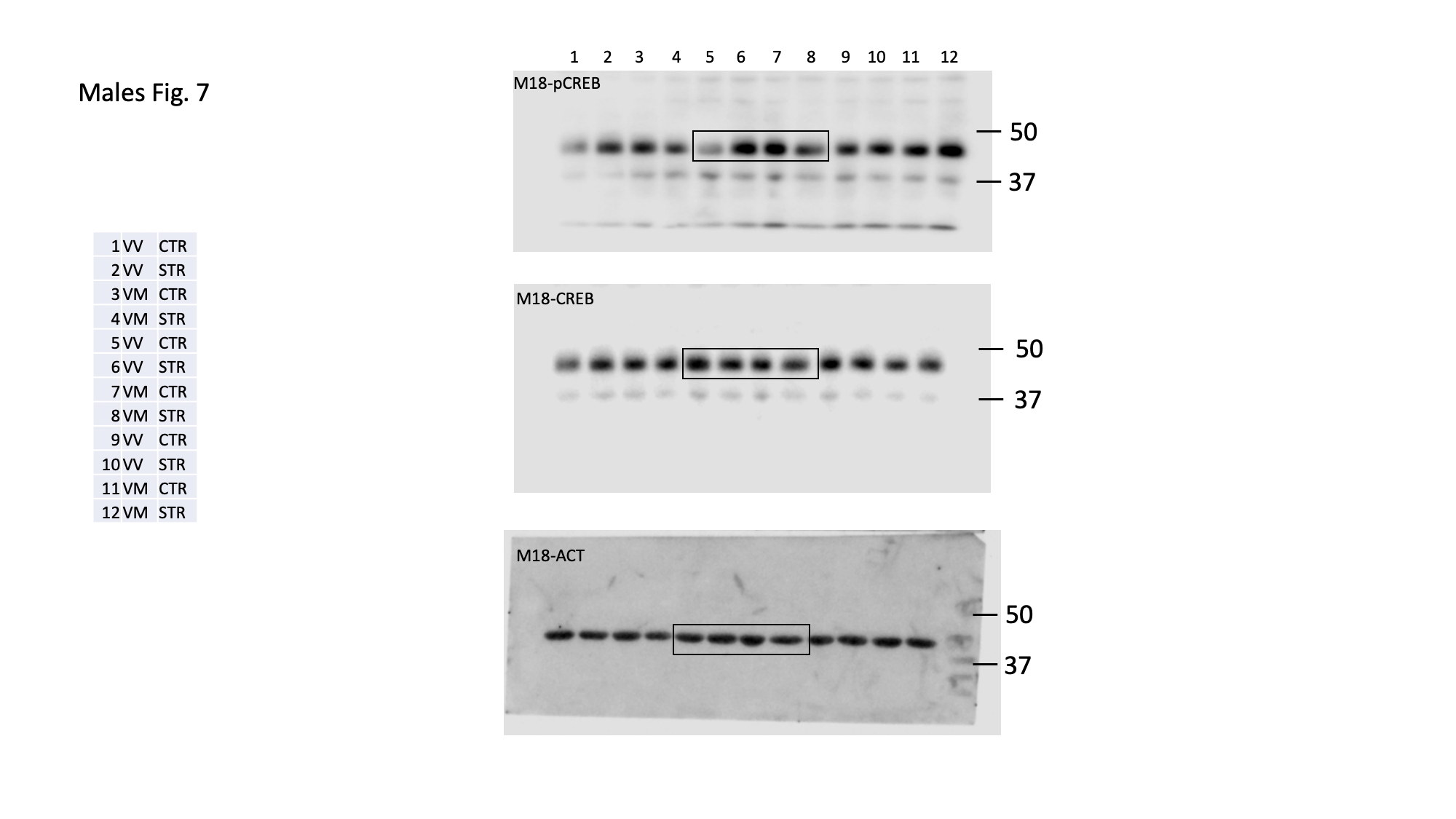

Supplement: Supplementary file 1 [file biology-13-00303-s001.zip › Fig7_pCREB-CREB_Males.tiff]
